# Genetic Polymorphism of *CYP2R1*, *CYP27A1*, *CYP27B1*, and Vitamin D Metabolites Plasma Levels in Patients with Cardiovascular Disease: A Pilot Study

**DOI:** 10.3390/biom15050699

**Published:** 2025-05-11

**Authors:** Mohamed Abouzid, Łukasz Kruszyna, Dominika Kaczmarek, Leonid Kagan, Aniceta Ada Mikulska-Sauermann, Dorota Filipowicz, Matylda Resztak, Franciszek K. Główka, Marta Karaźniewicz-Łada

**Affiliations:** 1 Department of Physical Pharmacy and Pharmacokinetics, Poznan University of Medical Sciences, Rokietnicka 3, 60-806 Poznan, Poland; mmahmoud@ump.edu.pl (M.A.); dominika.kamk@gmail.com (D.K.); amikulska@ump.edu.pl (A.A.M.-S.); mresztak@ump.edu.pl (M.R.); glowka@ump.edu.pl (F.K.G.); 2 Doctoral School, Poznan University of Medical Sciences, Bukowska 70, 60-812 Poznan, Poland; 3 Department of Vascular and Endovascular Surgery, Angiology and Phlebology, Poznan University of Medical Sciences, Długa ½, 60-848 Poznan, Poland; lukaszkruszyna@ump.edu.pl; 4 Department of Pharmaceutics, Center of Excellence for Pharmaceutical Translational Research and Education, Ernest Mario School of Pharmacy, Rutgers, the State University of New Jersey, Piscataway, NJ 0885, USA; lkagan@pharmacy.rutgers.edu; 5 Department of Endocrinology, Metabolism and Internal Medicine, Poznan University of Medical Sciences, 49 Przybyszewskiego Street, 60-355 Poznan, Poland; dorota.filipowicz123@gmail.com

**Keywords:** *CYP2R1*, *CYP27A1*, *CYP27B1*, calcitriol, calcidiol, cardiovascular disease

## Abstract

The active form of vitamin D, calcitriol (1,25(OH)_2_D_3_), is produced from 25(OH)D_3_ via enzymes encoded by *CYP2R1*, *CYP27A1*, and *CYP27B1*. Polymorphisms in these genes may alter vitamin D metabolism and increase cardiovascular disease risk. This preliminary study investigated these polymorphisms in 27 patients with cardiovascular disease and 26 healthy volunteers using Polymerase Chain Reaction—Restriction Fragment Length Polymorphism (PCR-RFLP), while measuring 25(OH)D_3_ and 1,25(OH)_2_D_3_ concentrations by UPLC-MS/MS and ELISA, respectively. Among patients, those with the GT genotype of *rs10877012* (*CYP27B1*) had higher 25(OH)D_3_ levels compared to other genotypes. Additionally, this polymorphism was associated with lower 1,25(OH)_2_D_3_ in TT homozygotes, suggesting reduced *CYP27B1* activity. Furthermore, the TT genotype of *rs6709815* (*CYP27A1*) was three times more prevalent in cardiac patients than in healthy controls, possibly indicating increased susceptibility to the disease. Although these findings suggest a genetic influence on vitamin D metabolism in cardiovascular disease, larger and more comprehensive studies are needed to confirm these associations.

## 1. Introduction

Cardiovascular disease (CVD) is a diagnosis of several heart and blood vessel diseases, including coronary artery disease, rhythm disturbances, valvular heart disease, carotid artery disease, congestive cardiomyopathy, and cerebrovascular accidents such as stroke [1]. CVD remains the leading cause of death globally, and millions of lives are lost prematurely to heart disease each year [2]. In Poland, CVD, including ischemic heart disease and stroke, is the leading cause of death, accounting for more than one-third of all deaths [3]. Approximately 1.4 million Polish citizens have heart failure, and ischemic heart disease mortality was more than double compared to other European Union countries, with 131 deaths per 100,000 [4].

CVD arises from a combination of factors, including socioeconomic status and access to healthcare [5], psychosocial factors [6], personal behaviors such as diet, physical activity, and smoking [7,8], environmental influences like air pollution [9,10], and genetic predisposition [11]. These elements interact in a complex way, increasing the risk of developing heart disease and related conditions [5]. Recent genetic studies have identified over 100 single-nucleotide polymorphisms (SNPs) associated with an increased risk of CVD [12]. However, the exact mechanisms by which these SNPs influence the development and progression of CVD remain unclear [13].

Since the early 20th century, vitamin D has been recognized for its pivotal role in the prevention and treatment of bone diseases such as rickets and osteomalacia [14]. While its primary functions involve maintaining calcium and phosphate homeostasis, growing evidence suggests that vitamin D is intricately linked to cardiovascular health and disease [15,16]. This connection is rooted in the transcriptional activity of vitamin D, which influences the expression of approximately 3% of the human genome, including genes involved in cardiovascular regulation [17].

Numerous observational studies have highlighted a significant association between vitamin D deficiency and various cardiovascular conditions [18,19]. These include arterial stiffness, hypertension, atherosclerosis, and left ventricular hypertrophy, all of which contribute to an increased risk of cardiovascular morbidity and mortality [18,20,21]. Vitamin D is believed to exert its effects through multiple mechanisms, such as improving endothelial function, supporting cardiomyocyte health, and modulating the calcification processes in valves and vascular tissues [22,23,24,25]. Notably, the variability across studies and clinical trials investigating the role of vitamin D in cardiovascular health poses substantial challenges to reaching definitive conclusions and establishing universal recommendations [15,18,19].

**Figure 1:**
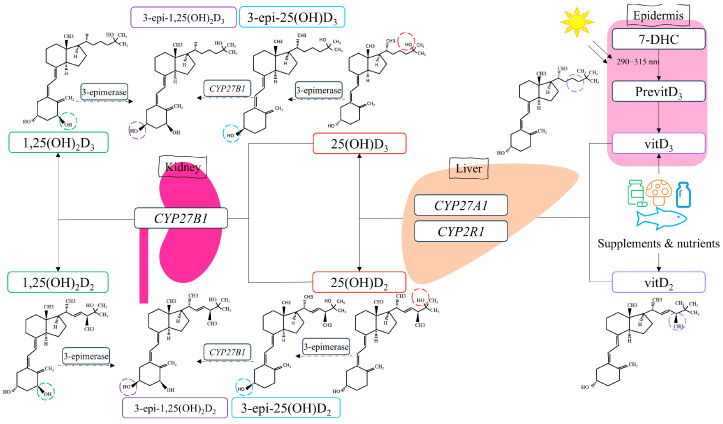
Vitamin D metabolism. Vitamin D_3_ is primarily synthesized in the skin through exposure to ultraviolet B radiation from sunlight and can also be obtained from dietary sources such as salmon, fortified milk, and vitamin D supplements. Vitamin D_2_ is mainly derived from plant-based sources, including mushrooms exposed to UV light, and is also available through dietary supplements. Both forms undergo the first hydroxylation process in the liver, catalyzed mainly by the enzyme CYP2R1 and, to a lesser extent, CYP27A1, resulting in the formation of 25(OH)D_3_ and 25(OH)D_2_. These 25-hydroxyvitamin D forms are then hydroxylated in the kidney by *CYP27B1* to produce the active forms, 1α,25(OH)_2_D_3_ and 1α,25(OH)_2_D_2_, with *CYP27B1* exhibiting a stronger affinity for 25(OH)D_3_. Epimerization by 3-epimerase can convert either 25(OH)D_3_ to 3-epi-25(OH)D_3_ or 25(OH)D_2_ to 3-epi-25(OH)D_2_. These 3-epi forms can further undergo additional epimerization and hydroxylation by *CYP27B1* to yield 3-epi-1α,25(OH)_2_D_3_ or 3-epi-1α,25(OH)_2_D_2_, respectively, depending on the substrate. The figure also illustrates the changes occurring in metabolite structures. Rectangles of different colors correspond to circles on the related metabolite structures, highlighting the structural differences that emerge after each process.

The mechanism of action for vitamin D involves binding the active metabolite, calcitriol, to the vitamin D receptor, which belongs to the steroid hormone family of nuclear receptors accountable for the transcriptional regulation of several hormone-responsive genes [26]. However, calcitriol is synthesized through metabolic reactions involving several enzymes regulated by vitamin D metabolism genes such as *CYP2R1*, *CYP27A1*, and *CYP27B1* (Figure 1).

Our previously published comprehensive review highlighted how the polymorphisms in these genes could influence several disorders [27]. However, information on the association between *CYP2R1*, *CYP27A1*, and *CYP27B1* variants and CVD is limited. According to Wang et al., the presence of *CYP2R1-rs10741657* significantly increases the risk of coronary artery disease in men over 60 years old [28]. This relationship was also observed in studies by Hassanein et al., where the GG genotype was associated with a 6.22-fold higher risk of coronary artery disease than the AA genotype [29]. Studies in the Polish population, however, showed lower estimated glomerular filtration rate values in AA homozygotes for the *CYP27B1-rs10877012* polymorphism, indicating impaired kidney function and a risk factor for left ventricular heart failure [30]. For *CYP27A1-rs4674344*, carriers of the T allele were found to have a higher risk of developing metabolic syndrome due to disruptions in the levels of pro-inflammatory adipocytokines [31].

The analyses conducted to date have not provided a conclusive understanding of the role of gene polymorphisms related to vitamin D metabolism in CVD, highlighting the need for further investigation. Our study aims to quantify vitamin D hydroxy metabolites (25(OH)D_2_ and 25(OH)D_3_, their epimers 3-epi-25(OH)D_2_ and 3-epi-25(OH)D_3_, and the active form 1,25(OH)_2_D_3_) in patients with CVD and healthy volunteers. We also identified polymorphisms in the *CYP2R1*, *CYP27A1*, and *CYP27B1* genes and evaluated their impact on plasma vitamin D metabolite concentrations. To our knowledge, no similar studies have been conducted.

## 2. Materials and Methods

### 2.1. Study Population

The study involved 27 adult patients with cardiovascular diseases treated at the Department of Vascular Surgery, Endovascular Surgery, Angiology, and Phlebology of Poznan University of Medical Sciences in Poznań. Inclusion criteria were as follows: age over 18 years, male or female, diagnosis and/or treatment of CVD prior to admission. Exclusion criteria were as follows: supplementation with vitamin D, acute myocardial infarction, a positive history of cancer, impaired renal function determined by serum creatinine concentration >2 mg/dL, and current liver dysfunction.

The patients had venous thrombosis (*n* = 9), atherosclerosis of the lower extremities (*n* = 14), atrial fibrillation (*n* = 19), hypertension (*n* = 13), and coronary artery disease (*n* = 5). Other comorbidities were diabetes (*n* = 7) and respiratory conditions such as bronchial asthma and chronic obstructive pulmonary disease (*n* = 5). Patients were treated with rivaroxaban (*n* = 27), statins (*n* = 14), acetylsalicylic acid (*n* = 11), clopidogrel (*n* = 4); other medications were prescribed individually. Recruitment was ongoing throughout the year from December 2021 to January 2025.

The healthy volunteer group comprised 26 healthy adults recruited from the Department of Endocrinology, Metabolism, and Internal Diseases at the University Clinical Hospital in Poznań. Inclusion criteria were as follows: age over 18 years, male or female, and lack of thyroid disease confirmed by biochemical and imaging tests. Exclusion criteria were any diagnosed disease prior to admission, and poor health according to physical examination and laboratory analyses. Recruitment was from 2023 to 2024.

Blood samples were collected and stored at −80 °C until genetic analysis. For UPLC-MS/MS analysis, plasma was separated by centrifuging the blood samples at 1620 g for 10 min. The separated plasma was then stored at −80 °C until further analysis.

The study was planned and conducted according to the ethical principles of the Declaration of Helsinki [32]. The study received approval from the Bioethics Committee of the Poznań University of Medical Sciences (approval numbers: 873/19, 58/20, 201/21, and 510/21).

### 2.2. Determining Concentrations of Vitamin D Hydroxy Metabolites in Plasma

We used our validated UPLC-MS/MS method to measure the concentrations of 25(OH)D_2_, 25(OH)D_3_, 3-epi-25(OH)D_2_, and 3-epi-25(OH)D_3_ [33]. Briefly, 200 μL of patient plasma was mixed with 20 μL of methanol and 20 μL of the internal standard, D6-25(OH)D_3_. The analytes were extracted using two 1000 μL portions of hexane. After evaporation, the residue was re-dissolved in 200 μL of a methanol–water (80:20, *v*/*v*) solution, and 10 μL was injected directly into a UPLC Nexera coupled with a triple quadrupole mass spectrometer LCMS-8030 (Shimadzu, Kyoto, Japan). The separation of the analytes was carried out using a Kinetex 2.6 μm F5 analytical column (50 mm × 2.1 mm) (Phenomenex, Torrance, CA, USA) with a mobile phase of methanol–water (80:20, *v*/*v*) containing 0.1% formic acid. The MS detection was performed in positive electrospray ionization mode. The specific *m*/*z* transitions for the analytes and chromatograms were provided as the Supplementary Material (Table S1, Figure S1). The assay was linear from 2 to 100 ng mL⁻^1^ (r^2^ ≥ 0.995). The LOD and LOQ were 1 ng/mL and 2 ng/mL, respectively. The recovery of the analytes from plasma samples ranged from 70.1% to 97.1%. The precision, both inter- and intra-day, varied between 2.1% and 18.1%, and the accuracy of the method expressed by the relative error was between 0.9% and 14.7%.

Determination of 1,25(OH)_2_D_3_ was conducted by ELISA kit [DHVD_3_ (1,25-Dihydroxyvitamin D_3_) Competitive ELISA (St. John’s Laboratory, London, UK)] in accordance with the procedure described by the manufacturer. The kit has an identification range of 15.625–1000 pg/mL.

### 2.3. Genetic Analyses

The isolation of the genomic DNA from 200 μL of blood was performed using a commercially available kit according to the manufacturer’s instructions (GenMATRIX Quick Blood Purification Kit, EURx, Poland). To identify *CYP2R1*, *CYP27A1*, and *CYP27B1* polymorphisms, polymerase chain reaction (PCR) coupled with restriction fragment length analysis (RFLP) was performed. Primers were used, as reported by Stjepanovic et al. [34], Giray [35], and Latacz et al. [36], respectively. Their sequences were checked by the Primer3Plus software version: 3.3.0 [37]. The primers’ sequences, their annealing temperatures, the length of the products obtained, and the results of restriction are presented in the Supplementary Material (Table S2).

### 2.4. Statistical Analysis

Excel (Microsoft Corp., Redmond, WA, USA) and Statistica 13.3 (StatSoft Inc., Tulsa, OK, USA) were used for statistical analysis. The Shapiro-Wilk test was applied to assess the normality of the variables. Normally distributed data are presented as mean ± SD, while non-normally distributed data are reported as median and interquartile range. A Student’s *t*-test was used for two groups with normal distribution, and ANOVA for three groups to compare the genotypes of *CYP2R1*, *CYP27A1*, and *CYP27B1*. For non-normal distributions, the Mann-Whitney U test and Kruskal-Wallis ANOVA were applied. The χ^2^ test assessed allele frequencies’ adherence to Hardy–Weinberg equilibrium, with a critical value of 3.84 (for *n* = 1 and α = 0.05). We also performed a logistic regression for the predictors of CVD and reported the results as odds ratios (ORs) and 95% confidence intervals (CIs). We considered *p*-value < 0.05 statistically significant for all tests.

## 3. Results

### 3.1. General Characteristics

We included 27 adult patients with CVD (*n* = 27) (fourteen males and thirteen females, aged 68 (63–73) years) and 26 healthy volunteers (twenty-five females and one male, aged 35.5 (27–41) years) (Table 1).

The distribution of *CYP2R1*, *CYP27A1*, and *CYP27B1* genotypes in the studied groups was consistent with Hardy–Weinberg equilibrium (χ2 < 3.84, α = 0.05). Allele frequencies of the polymorphisms in the studied population and healthy volunteers are presented in Table 2.

**Figure 2:**
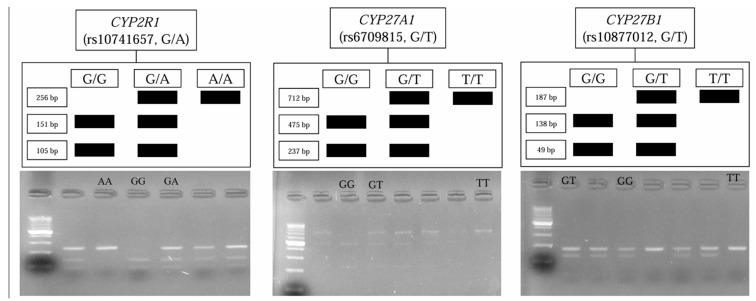
Genotyping of *CYP2R1*, *CYP27A1,* and *CYP27B1* by PCR-RFLP. (**Top row**) Schematic diagrams showing the expected restriction-fragment sizes for each genotype. (**Bottom row**) Representative agarose-gel images from selected study participants; each gel displays all three genotypes for the corresponding SNP. Fragment patterns match the schematics above (e.g., *CYP2R1* GA: 276 bp + 145 bp + 131 bp).

The comparison shows that these frequencies are statistically similar. However, significant differences were observed in the frequency of the TT genotype, with TT homozygotes comprising 40% of patients with CVD compared to 13.6% of healthy volunteers. Sample of *CYP2R1*, *CYP27A1*, and *CYP27B1* genotypes are shown in Figure 2.

### 3.2. CYP2R1 (rs10741657)

No significant differences were observed in anthropometric parameters or vitamin D metabolite concentrations based on the *rs10741657* polymorphism among patients (Table 3) or healthy volunteers (Table 4).

### 3.3. CYP27A1 (rs6709815)

No significant differences were observed in any analyzed parameters based on this polymorphism for patients (Table 5).

For healthy volunteers, statistically significant differences were observed in the 3-epi-25(OH)D_3_ percentage in the total 25(OH)D_3_ concentration. In carriers of the GT genotype, the epimeric form of 25(OH)D_3_ accounted for 10.7% of the total 25(OH)D_3_ concentration, while in GG and TT homozygotes, it was twice as high (Table 6).

### 3.4. CYP27B1 (rs10877012)

**Figure 3:**
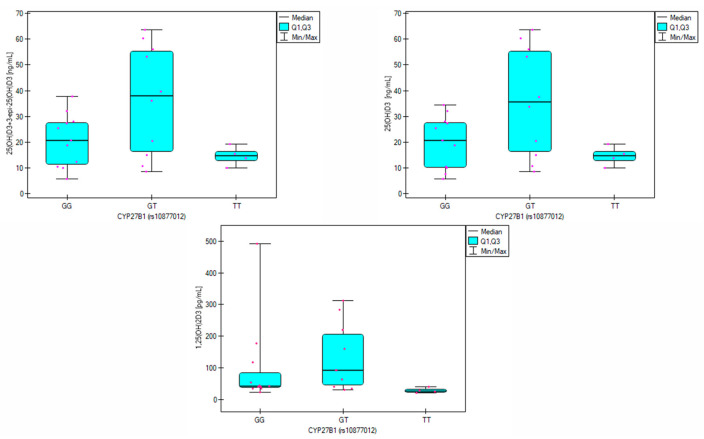
Significant differences in the concentrations of 25(OH)D_3_, 1,25(OH)_2_D_3_, and 25(OH)D_3_ + 3-epi-25(OH)D_3_ across *CYP27B1* (*rs10877012*) genotypes in patients with cardiovascular disease. Individual patient values are shown as purple dots.

Significant differences in 25(OH)D_3_ and its epimer concentrations were observed across genotypes for patients (Figure 3).

Individuals with the GT genotype had higher mean calcidiol levels (35.9 ± 21.4 ng/mL) when compared with carriers of GG and TT genotypes (20.1 ± 10.2 ng/mL and 14.7 ± 3.9 ng/mL, respectively). GT individuals also showed the highest total concentrations of 25(OH)D_3_ and its epimer, whereas TT genotype individuals had the lowest parameter value. Differences in 1,25(OH)_2_D_3_ concentrations were also noted, with TT individuals exhibiting lower calcitriol levels than GG and GT individuals (Table 7).

Significant differences in 25(OH)D_2_ concentrations were observed for healthy volunteers based on genotype. Individuals with the TT genotype had an average concentration of 13.9 ± 0.8 ng/mL, nearly double that of the other genotypes. Additionally, the sum of 25(OH)D_2_ and its epimer concentrations was higher in TT homozygotes, with this difference being statistically significant at the trend level. Higher calcitriol levels in TT individuals were also observed, approaching statistical significance (Table 8).

### 3.5. Predictors of CVD

The CYP27A1-TT genotype was near the significance threshold with an OR of 4.22 (95% CI: 0.983–18.127, *p* = 0.053). The pooled univariate analysis results are shown in Table 9.

## 4. Discussion

In the present study, we analyzed the *CYP2R1*, *CYP27A1*, and *CYP27B1* genetic polymorphisms and their association with anthropometric parameters, concentrations of 25(OH)D_2_, 25(OH)D_3_, 3-epi-25(OH)D_2_, 3-epi-25(OH)D_3_, and 1,25(OH)_2_D_3_, in Polish patients with cardiovascular disease and healthy subjects. To quantify vitamin D metabolites, we used the validated UPLC-MS/MS method, which is considered the gold standard technique for analyzing vitamin D status. This method is suitable for the simultaneous detection of multiple vitamin D metabolites and is not subject to matrix interference, unlike immunoassays [38]. We found that the genetic distributions of *CYP2R1*, *CYP27A1*, and *CYP27B1* genotypes aligned with Hardy–Weinberg equilibrium, and allele frequencies were generally similar to the general Polish population, though the TT genotype was significantly more common in thrombosis patients (40%) than in healthy individuals (13.6%). No significant associations were observed between the *rs10741657 CYP2R1* polymorphism and anthropometric or vitamin D metabolite levels. For the *rs6709815 CYP27A1* polymorphism, no significant differences were seen in patients, but in healthy subjects, 3-epi-25(OH)D_3_ percentages varied by genotype. Analysis of the *rs10877012 CYP27B1* polymorphism revealed significant genotype-based differences in vitamin D metabolite concentrations, with GT individuals showing the highest 25(OH)D_3_ and calcitriol levels, while in TT individuals the values were the lowest. In the healthy volunteers, the TT genotype was associated with significantly higher 25(OH)D_2_ concentrations and a trend toward higher calcitriol levels.

In regard to 25(OH)D_3_ levels, in both groups, the majority of individuals had concentrations below the optimal value of 30 ng/mL, with the average value being numerically lower in patients with CVD (20.4 ng/mL) than in healthy individuals (25.5 ng/mL). Vitamin D insufficiency (25(OH)D_3_ concentration < 20 ng/mL) was found in 44.4% of patients and 34.6% of healthy volunteers, indicating the prevalence of vitamin D deficiency in the analyzed population. Oberoi et al. also demonstrated differences in calcidiol levels between individuals with CVD and healthy individuals, whose study among Indian residents showed vitamin D deficiency (using a different classification, serum level < 30 ng/mL) in 63% of sick individuals and 35% of healthy controls [39]. Lower concentrations in the study group compared to the healthy volunteers’ group were also observed for epimeric forms of vitamin D. The average 3-epi-25(OH)D_3_ concentration was 2.4 ng/mL in the study group and 3.7 ng/mL in the control group, while for 3-epi-25(OH)D_2_, the average concentration in the study group was below the lower quantification limit of the method (<2.0 ng/mL), and in the group of healthy volunteers, it was 5.1 ng/mL. Meanwhile, 25(OH)D_2_ concentrations were higher in patients with CVD (8.1 ng/mL) compared to healthy individuals (6.5 ng/mL). Differences in 1,25(OH)_2_D_3_ concentrations were also noted between the two groups, with patients having an average calcitriol concentration of 43.4 pg/mL, compared to 67.9 pg/mL in healthy individuals. However, none of the differences in metabolite concentrations were statistically significant (Table 1).

For the *rs10741657 CYP2R1* polymorphism, both groups’ genotype and allele frequencies were consistent with Hardy–Weinberg equilibrium. A comparison of genotype frequencies between the study group and the healthy volunteers group showed no statistically significant differences, so there is no basis to suggest an association between the *rs10741657* polymorphism and CVD in the analyzed population. In a study involving German patients with type 2 diabetes and healthy controls, Klahold et al. [40] also reported that allele frequencies of *rs10741657* were in Hardy–Weinberg equilibrium. However, they noticed that the “A” allele of *CYP2R1 rs10741657* was more frequent among patients (42.1% vs. 36.3%; OR: 1.28; CI: 1.07–1.53; *p* = 0.016). Our study did not observe such a relationship, even though it was numerically higher in the patient group than in healthy volunteers (60% vs. 40%; OR: 2.33; CI: 0.78–7.09; *p* = 0.132).

Vitamin D metabolite concentrations did not differ significantly based on genotype, either among patients or healthy individuals (Table 3 and Table 4). In patients, the lowest 25(OH)D_3_ concentration was found in AA homozygotes, with an average of 15.02 ng/mL, while the highest average concentration was observed in heterozygotes (22.4 ng/mL). Similar results were obtained in individuals without CVD, where the lowest average 25(OH)D_3_ concentration occurred in volunteers with the AA genotype (21.3 ng/mL) and the highest in GA heterozygotes (26.4 ng/mL). Therefore, this study could not confirm the findings of Duan et al. in the European population [41] or Hassanein et al. in the Egyptian population [29], which indicated that the GG genotype is associated with lower 25(OH)D levels. The discrepancy may be due to the small sample size or ethnic differences between populations.

For the *rs6709815 CYP27A1* polymorphism, genotype and allele frequencies among patients and healthy individuals remained in Hardy–Weinberg equilibrium. In the group of individuals with CVD, the T allele was less frequent (T = 0.620) compared to the values defined for the Turkish patients with multiple sclerosis (T = 0.816) [35], while this allele in our results was less frequent among healthy individuals (T = 0.432). Furthermore, significant differences were observed in the frequency of the *TT* genotype between the analyzed groups (*p* = 0.044). TT homozygotes were over three times more frequent among individuals with thrombosis than in healthy individuals, as shown in Table 2. To date, there have been no scientific reports on the *rs6709815* polymorphism as a risk factor for CVD. However, the results of this study indicate a potential association between this polymorphism and CVD.

No significant differences in vitamin D metabolite concentrations were observed in the study group based on genotype. In contrast, in the group of healthy volunteers, significant differences were found in the percentage of the epimeric form of 25-hydroxyvitamin D_3_ in the total 25(OH)D_3_ pool (*p* = 0.0289). The lowest percentage of 3-epi-25(OH)D_3_, amounting to 10.3%, was found in individuals with the *CYP7A1-GT* genotype, while for the GG and TT genotypes, the percentages were 22.0% and 20.0%, respectively (Table 6). To date, no studies have demonstrated a relationship between the *rs6709815* polymorphism and changes in vitamin D metabolite concentrations in the body [42].

The frequency analysis of the *rs10877012 CYP27B1* polymorphism showed that the gene variation remained in Hardy–Weinberg equilibrium. Allele frequencies in both analyzed groups were similar to those observed in the pan-European population [43]. The genotypes occurred with similar frequencies in both the study group and the group of healthy volunteers (Table 2), so no association between the *rs10877012* polymorphism and CVD was identified in the analyzed population. However, significant differences in vitamin D metabolite levels were observed depending on the genotype in the patient group. In heterozygotes, the average 25(OH)D_3_ concentration was 35.9 ng/mL, while in GG and TT homozygotes, it was 20.06 ng/mL and 14.66 ng/mL, respectively (*p* = 0.034, Table 7). This is consistent with the findings of Meirina et al., who analyzed 25(OH)D_3_ levels in pregnant women and identified the highest levels in those with the GT genotype and the lowest in TT homozygotes. In this group, the correlation between the G allele and reduced vitamin D levels was significant at a statistical trend level (*p* = 0.057) [44].

Additionally, Hu et al. observed the best vitamin D supplementation results in individuals with the GT genotype [45]. Significant differences in 1,25(OH)_2_D_3_ levels were also noted among patients (*p* = 0.032), with the highest concentrations observed for the GT genotype (92.9 pg/mL) and lower for GG (43.4 pg/mL) and TT (28.1 pg/mL). Post-hoc analysis showed that 1,25(OH)_2_D_3_ levels were significantly lower in individuals with the TT genotype than the GT genotype (*p* = 0.027). *CYP27B1* is the enzyme responsible for 1α-hydroxylation of 25(OH)D_3_ to calcitriol. Therefore, these results suggest that the TT genotype may reduce the enzymatic activity of *CYP27B1*. In the group of healthy volunteers, statistically significant differences were observed in 25(OH)D_2_ concentrations depending on genotype (*p* = 0.043). In the case of TT homozygotes, the 25(OH)D_2_ level was the highest and amounted to 13.9 ng/mL, while in the case of heterozygotes and GG homozygotes, it was 7.9 ng/mL and 6.3 ng/mL, respectively (Table 8). Differences in calcitriol concentrations were also observed, with healthy individuals with the TT genotype having an average concentration of 259.4 pg/mL, while in individuals with the GT and GG genotypes, the concentrations were 57.6 pg/mL and 66.1 pg/mL, respectively.

However, it is essential to note that vitamin D metabolism in the body is a complex process, so other factors not analyzed in this study may contribute to the differences in metabolite levels. Nevertheless, identifying the *rs10877012 CYP27B1* genotype may help determine appropriate vitamin D supplementation doses for individuals with CVD.

## 5. Limitations

Our study is the first to report the genetic distributions of *CYP2R1*, *CYP27A1*, and *CYP27B1* genotypes and their associations with vitamin D hydroxy metabolites, including 25(OH)D_2_, 25(OH)D_3_, 3-epi-25(OH)D_3_, 3-epi-25(OH)D_2_, and calcitriol, in healthy Polish volunteers and patients with CVD. However, it is essential to acknowledge the limitations of this study. Firstly, we did not account for seasonal variations, which could have influenced metabolite levels—seasonal analysis was performed in our previous observational study [46]. Secondly, there was a notable age difference between participants with CVD (median 68 years) and healthy participants (median 35.5 years), primarily due to the challenge of recruiting older individuals without cardiovascular conditions. Therefore, splitting the results reporting for healthy volunteers and patients was also essential. Third, *rs6709815-CYP27A1* could not be identified in two patients and four healthy group members. Hence, despite the chi-square analysis indicating statistical significance (*p* < 0.05) and the *CYP27A1-TT* genotype approaching the threshold for statistical significance, with an odds ratio of 4.22 (95% CI: 0.983–18.127; *p* = 0.053), this result should be considered a proof of concept rather than proof of evidence.

Fourth, we did not adjust *p*-values for multiple comparisons because we aimed to explore each genotype separately. However, we acknowledge that this approach may increase the risk of Type I errors, and findings should be interpreted cautiously. Lastly, we did not collect data on patient behaviors and lifestyle factors that may influence vitamin D status. For instance, supplementation patterns or attitudes among healthy volunteers were not identified for all participants. Consequently, the findings of this study should not be generalized and interpreted with caution until confirmed by a larger sample size, because only 53 participants were enrolled; this work should be regarded as a pilot study.Post-hoc power for the three target SNPs did not exceed 25%. A prospective calculation (α = 0.05, 1-β = 0.80, OR = 2.0) and minor allele frequency (rs6709815, 0.47 [47]; rs10741657, 0.37 [48]; and rs10877012, 0.47 [36]) show that 135–155 cases and an equal number of controls would be required for a definitive study.

## 6. Conclusions

Individuals with the TT genotype of *rs6709815* *CYP27A1* may have an increased risk of developing CVD compared to other genotypes. Additionally, the presence of the TT genotype of *rs10877012 CYP27B1* is associated with lower concentrations of 1,25(OH)_2_D_3_ in individuals with CVD, suggesting that this genotype may result in reduced enzymatic activity of CYP27B1. Finally, the *rs10741657 CYP2R1* polymorphism does not influence the plasma concentrations of vitamin D metabolites in both patients with CVD and healthy individuals. Our results require confirmation through a larger study, particularly those related to *rs6709815*-TT and CVD. Establishing the relationship between genetic polymorphisms and vitamin D metabolite levels may help in identifying individuals at increased risk of vitamin D deficiency. Particularly, the identification of the *rs10877012* genotype may be helpful in determining the appropriate vitamin D supplementation protocol for individuals with cardiovascular diseases.

## Figures and Tables

**Table 1:** Baseline characteristics of patients and volunteers [values given as number of patients *n* (%), mean (SD) or median (IQR)].

Parameter	Patients	Healthy Volunteers	*p*
Number	27	26	
Females	13 (48.1%)	25 (96.2%)	<0.001 ^†^
Age [year]	68.0 (63.0–73.0)	35.5 (27.0–41.0)	<0.001
Weight [kg]	79.5 (65.0–92.0)	71.65 (63.7–82.8)	0.47
Height [cm]	169.7 ± 8.2	166.7 ± 7.4	0.092
BMI [kg/m^2^]	27.0 ± 4.3	26.7 ± 5.2	0.849
25(OH)D_3_ [ng/mL]	20.4 (10.7–33.7)	25.51 (16.2–30.6)	0.421
3-epi-25(OH)D_3_ [ng/mL]	2.4 ± 0.5	3.67 ± 1.46	0.062
25(OH)D_2_ [ng/mL]	8.1 (7.8–8.4)	6.46 (4.5–13.4)	0.557
3-epi-25(OH)D_2_ [ng/mL]	<2.0 *	5.1 ± 2.2	
25(OH)D_3_ + 3-epi-25(OH)D_3_ [ng/mL]	20.4 (12.3–36.2)	26.2 (17.7–31.9)	0.235
3-epi-25(OH)D_3_ [%]	12.0 ± 7.4	13.8 ± 5.8	0.675
25(OH)D_2_ + 3-epi-25(OH)D_2_ [ng/mL]	8.1 (7.8–8.4)	10.1 (6.2–13.4)	0.484
3-epi-25(OH)D_2_ [%]		37.99 (36.2–41.9)	
1,25(OH)_2_D_3_ [pg/mL]	43.4 (36.0–117.5)	67.9 (35.4–157.2)	0.458

* Concentrations below the lower limit of quantification for the applied UPLC-MS/MS method (LLOQ = 2.00 ng/mL). ^†^ *p*-value of Chi-square.

**Table 2:** Allele frequencies of the *CYP2R1*, *CYP27A1*, and *CYP27B1* polymorphisms in patients and healthy volunteers, reported as *n* (%).

	Patients	Healthy Volunteers	*p*
*rs10741657 CYP2R1*			
GG	9 (33.3)	14 (53.8)	0.132
GA	15 (55.6)	10 (38.5)	0.213
AA	3 (11.1)	2 (7.7)	0.670
*rs6709815 CYP27A1* *			
GG	4 (16)	6 (27.3)	0.356
GT	11 (44)	13 (59.1)	0.302
TT	10 (40)	3 (13.6)	0.044
*rs10877012 CYP27B1*			
GG	13 (48.1)	10 (38.5)	0.477
GT	10 (37)	13 (50)	0.341
TT	4 (14.8)	3 (11.5)	0.725

* The genotype could not be identified in two patients and four healthy group members, so they were excluded from the analysis of this polymorphism.

**Table 3:** Patient characteristics according to the rs10741657 polymorphism.

Parameter	GG (*n* = 9) ^†^	GA (*n* = 15)	AA (*n* = 3) ^†^	*p*
Weight [kg]	91.0 (86.0–94.0)	70.0 (65.0–82.0)	70.0 (60.0–80.0)	0.229
BMI [kg/m^2^]	30.8 (29.1–31.1)	25.9 (22.9–28.4)	25.4 (22.9–28.0)	0.233
25(OH)D_3_ [ng/mL]	20.6 (17.2–41.6)	22.4 (10.4–33.7)	15.0 (7.7–37.6)	0.691
3-epi-25(OH)D_3_ [ng/mL]	2.18	2.83 (2.5–3.2)	2.1 (2.1–2.2)	0.191
25(OH)D_2_ [ng/mL]	8.4 (8.3–9.0)	5.6 (3.4–7.8)	7.8	0.145
25(OH)D_3_ + 3-epi-25(OH)D_3_ [ng/mL]	20.6 (17.2–41.6)	22.4 (10.4–36.2)	15.0 (9.9–39.6)	0.655
3-epi-25(OH)D_3_ [%]	17.8	7.6 (6.8–8.5)	13.6 (5.2–22.0)	0.741
25(OH)D_2_ + 3-epi-25(OH)D_2_ [ng/mL]	8.4 (8.3–9.0)	5.6 (3.4–7.8)	7.8	0.145
1,25(OH)_2_D_3_ [pg/mL]	43.9 (34.2–115.9)	53.9 (34.6–117.5)	41.7 (36.0–282.9)	0.976

^†^ A single value indicates that only one patient had the metabolite’s concentration > LLOQ.

**Table 4:** Healthy volunteers’ characteristics according to the rs10741657 polymorphism.

Parameter	GG (*n* = 14)	GA (*n* = 10)	AA (*n* = 2) ^†^	*p*
Weight [kg]	74.6 (63.7–82.8)	72.1 (62.9–82.8)	68.9 (68.2–69.5)	0.922
BMI [kg/m^2^]	25.4 (22.3–31.9)	28.4 (22.8–30.1)	23.9 (23.9–24.1)	0.78
25(OH)D_3_ [ng/mL]	25.2 (16.2–29.7)	26.4 (16.8–31.9)	21.31 (4.8–37.8)	0.861
3-epi-25(OH)D_3_ [ng/mL]	2.7 (2.2–4.4)	3.9 (2.9–5.7)	2.9	0.52
25(OH)D_2_ [ng/mL]	6.5 (4.5–13.4)	7.1 (2.9–14.1)	5.9	0.817
3-epi-25(OH)D_2_ [ng/mL]	4.6 (2.2–8.5)	4.1 (3.8–7.6)	4.2	0.965
25(OH)D_3_ + 3-epi-25(OH)D_3_ [ng/mL]	25.9 (17.7–30.7)	26.4 (21.1–35.9)	27.8 (4.8–40.8)	0.893
3-epi-25(OH)D_3_ [%]	15.3 (8.9–18.9)	13.9 (10.7–14.3)	7.2	0.408
25(OH)D_2_ + 3-epi-25(OH)D_2_ [ng/mL]	6.5 (4.5–13.4)	11.2 (6.3–21.8)	10.1	0.836
3-epi-25(OH)D_2_ [%]	38.4 (36.2–53.3)	36.9 (35.2–37.9)	41.9	0.566
1,25(OH)_2_D_3_ [pg/mL]	67.5 (36.5–195.6)	47.8 (34.3–103.8)	93.6 (68.4–118.8)	0.758

^†^ A single value indicates that only one patient had the metabolite’s concentration > LLOQ.

**Table 5:** Patient characteristics according to the *rs6709815* polymorphism.

Parameter	GG (*n* = 4) ^†^	GT (*n* = 11)	TT (*n* = 10)	*p*
Weight [kg]	70.7 ± 11.0	82.8 ± 20.8	75.8 ± 14.7	0.517
BMI [kg/m^2^]	25.7 ± 2.8	28.5 ± 5.1	26.1 ± 3.8	0.438
25(OH)D_3_ [ng/mL]	12.1 (7.2–26.6)	19.3 (13.8–27.2)	33.7 (10.1–53.2)	0.359
3-epi-25(OH)D_3_ [ng/mL]	2.1	<2	2.3 (2.2–2.8)	1
25(OH)D_2_ [ng/mL]	8.4	5.6 (3.4–7.8)	8.4 (7.8–9.0)	0.497
25(OH)D_3_ + 3-epi-25(OH)D_3_ [ng/mL]	12.1 (7.2–27.6)	19.3 (13.8–27.2)	36.2 (12.3–53.2)	0.285
3-epi-25(OH)D_3_ [%]	5.2	<2	13.1 (7.6–19.9)	1
25(OH)D_2_ + 3-epi-25(OH)D_2_ [ng/mL]	8.4	5.6 (3.4–7.8)	8.4 (7.8–9.0)	0.497
1,25(OH)_2_D_3_ [pg/mL]	131.5 (41.8–356.3)	50.2 (34.6–160.9)	43.6 (36.0–92.9)	0.518

^†^ A single value indicates that only one patient had the metabolite’s concentration > LLOQ.

**Table 6:** Healthy volunteers’ characteristics according to the *rs6709815* polymorphism.

Parameter	GG (*n* = 6) ^†^	GT (*n* = 13)	TT (*n* = 3)	*p*
Weight [kg]	62.9 (54.5–73.8)	69.5 (64.5–82.8)	76.6 (61.4–80.1)	0.2594
BMI [kg/m^2^]	24.0 ± 3.9	26.5 ± 4.7	26.7 ± 4.9	0.515
25(OH)D_3_ [ng/mL]	27.9 ± 16.2	27.8 ± 14.8	17.9 ± 7.6	0.564
3-epi-25(OH)D_3_ [ng/mL]	5.7 (5.7–5.8)	2.9 (2.7–4.1)	3.5 (2.6–4.4)	0.255
25(OH)D_2_ [ng/mL]	9.8 (6.2–14.0)	6.5 (3.8–13.3)	5.0 (1.9–8.1)	0.492
3-epi-25(OH)D_2_ [ng/mL]	3.8	4.2 (3.8–5.9)	3.4 (2.2–4.6)	0.765
25(OH)D_3_ + 3-epi-25(OH)D_3_ [ng/mL]	29.9 ± 15.5	29.8 ± 15.9	20.3 ± 6.1	0.601
3-epi-25(OH)D_3_ [%]	22.0 (18.9–25.2)	10.7 (7.3–13.9)	20.0 (18.6–21.5)	0.029
25(OH)D_2_ + 3-epi-25(OH)D_2_ [ng/mL]	11.6 (8.0–14.0)	10.1 (6.3–13.3)	8.4 (4.1–12.7)	0.752
3-epi-25(OH)D_2_ [%]	37.9	39.5 (36.1–48.3)	44.8 (36.2–53.3)	1
1,25(OH)_2_D_3_ [pg/mL]	47.8 (30.5–218.6)	67.5 (42.8–100.7)	116.0 (36.5–195.6)	0.957

^†^ A single value indicates that only one patient had the metabolite’s concentration > LLOQ.

**Table 7:** Patient characteristics according to the *rs10877012* polymorphism.

Parameter	GG (*n* = 13)	GT (*n* = 10) ^†^	TT (*n* = 4) ^†^	*p*
Weight [kg]	90.0 (70.0–95.0)	70.0 (60.0–82.0)	69.0 (68.0–70.0)	0.247
BMI [kg/m^2^]	29.1 (24.2–31.1)	27.3 (22.9–28.4)	26.1 (25.7–26.6)	0.724
25(OH)D_3_ [ng/mL]	20.1 ± 10.2	35.9 ± 21.4	14.7 ± 3.9	0.034
3-epi-25(OH)D_3_ [ng/mL]	2.2 (2.2–3.2)	2.3 (2.1–2.5)	<2.0	1
25(OH)D_2_ [ng/mL]	8.4 (7.8–9.0)	8.34	7.4 (3.4–8.4)	0.807
25(OH)D_3_ + 3-epi-25(OH)D_3_ [ng/mL]	20.8 ± 10.3	36.4 ± 21.4	14.7 ± 3.9	0.033
3-epi-25(OH)D_3_ [%]	17.8 (8.5–22.0)	6.0 (5.2–6.8)	<2.0	1
25(OH)D_2_ + 3-epi-25(OH)D_2_ [ng/mL]	8.4 (7.8–90.1)	8.3	7.8 (3.4–8.4)	0.807
1,25(OH)_2_D_3_ [pg/mL]	43.4 (36.0–117.5)	92.9 (41.7–221.1)	28.1 (23.9–36.1)	0.032

^†^ A single value indicates that only one patient had the metabolite’s concentration > LLOQ.

**Table 8:** Healthy volunteers’ characteristics according to the *rs10877012* polymorphism.

Parameter	GG (*n* = 10)	GT (*n* = 13)	TT (*n* = 3)	*p*
Weight [kg]	77.6 ± 20.5	72.1 ± 10.6	72.1 ± 14.4	0.686
BMI [kg/m^2^]	28.0 ± 6.4	26.0 ± 4.2	25.6 ± 6.2	0.635
25(OH)D_3_ [ng/mL]	26.1 ± 12.2	25.9 ± 15.5	27.1 ± 12.8	0.992
3-epi-25(OH)D_3_ [ng/mL]	2.7 (2.2–4.4)	3.9 (3.4–4.9)	4.2 (2.2–6.2)	0.486
25(OH)D_2_ [ng/mL]	6.3 ± 3.4	7.9 ± 5.4	13.9 ± 0.8	0.043
3-epi-25(OH)D_2_ [ng/mL]	3.9 (2.9–4.4)	4.1 (3.4–7.7)	8.1 (7.6–8.5)	0.194
25(OH)D_3_ + 3-epi-25(OH)D_3_ [ng/mL]	28.4 ± 11.7	27.4 ± 15.9	29.9 ± 14.6	0.959
3-epi-25(OH)D_3_ [%]	11.9 (7.2–18.9)	12.3 (9.0–19.6)	14.1 (13.9–14.3)	0.826
25(OH)D_2_ + 3-epi-25(OH)D_2_ [ng/mL]	8.2 (4.3–11.4)	8.8 (6.2–14.6)	22.2 (13.3–22.4)	0.067
3-epi-25(OH)D_2_ [%]	39.9 (37.1–47.6)	36.9 (35.2–54.6)	36.2 (33.9–38.4)	0.638
1,25(OH)_2_D_3_ [pg/mL]	66.1 (30.5–76.2)	57.6 (36.5–118.8)	259.4 (103.8–429.9)	0.071

**Table 9:** Univariate genetic polymorphism predictors for CVD.

	B Coeff.	B Error	Wald Stat.	*p*-Value	Odds Ratio	−95% CI	+95% CI
*CYP2R1*-A ^†^	0.847	0.567	2.234	0.135	2.333	0.768	7.089
*CYP27A1*-T ^‡^	0.677	0.726	0.871	0.351	1.969	0.475	8.166
*CYP27B1*_T ^‡^	−0.396	0.558	0.504	0.478	0.673	0.226	2.007
*CYP2R1*-GA	0.693	0.559	1.537	0.215	2.000	0.669	5.982
*CYP2R1*-GG	−0.847	0.567	2.234	0.135	0.429	0.141	1.302
*CYP2R1*-AA	0.405	0.957	0.179	0.672	1.500	0.230	9.796
*CYP27A1*-GG	−0.677	0.726	0.871	0.351	0.508	0.122	2.107
*CYP27A1*-GT	−0.609	0.592	1.058	0.304	0.544	0.170	1.735
*CYP27A1*-TT	1.440	0.743	3.754	0.053	4.222	0.983	18.127
*CYP27B1*-GG	0.396	0.558	0.504	0.478	1.486	0.498	4.431
*CYP27B1*-GT	−0.531	0.559	0.901	0.343	0.588	0.197	1.760
*CYP27B1*-TT	0.288	0.819	0.123	0.725	1.333	0.268	6.635

^†^ with at least one “A” allele. ^‡^ with at least one “T” allele.

## Data Availability

The original contributions presented in this study are included in the article/Supplementary Material.

## Supplementary Materials

The following supporting information can be downloaded at: https://www.mdpi.com/article/10.3390/biom15050699/s1. Table S1. The ionic transitions of the analyzed compounds in MRM mode. Table S2. Primers’ sequences, annealing temperature, lengths of products obtained, and results of restriction in PCR reaction. Figure S1. Chromatograms for a blank sample, LOQ, and volunteer’s samples for the concentrations of (12 ng/mL, 2.9 ng/mL, 7 ng/mL, and 3.8 ng/mL for 25(OH)D3, 3-epi-25(OH)D3, 25(OH)D2, and 3-epi-25(OH)D2, respectively).

## Abbreviations

1,25(OH)_2_D_3_-calcitriol; 25(OH)D_2_-25-hydroxyvitamin D_2_; 25(OH)D_3_-25-hydroxyvitamin D_3_; 3-epi-25(OH)D_2_-3-epi-25-hydroxyvitamin D_2_; 3-epi-25(OH)D_3_-3-epi-25-hydroxyvitamin D_3_; CI-confidence interval; CVD-cardiovascular disease; LLOQ-lower limit of quantification; OR-odds ratio; PCR-RFLP-polymerase chain reaction–restriction fragment length polymorphism; SNPs-single-nucleotide polymorphisms; UPLC-MS/MS-ultra-performance liquid chromatography–tandem mass spectrometry.
