# Supplementary material for: Genetic Polymorphism of CYP2R1, CYP27A1, CYP27B1, and Vitamin D Metabolites Plasma Levels in Patients with Cardiovascular Disease: A Pilot Study
Source: Biomolecules. 2025 May 11;15(5):699. doi: 10.3390/biom15050699 (PMC12109444; doi:10.3390/biom15050699)

Table S1. The ionic transitions of the analyzed compounds in MRM mode.

Metabolite	Transitions. m/z		Q_1 [V]	CE [V]	Q_3 [V]
	Precursor ion	Product ion			
$25(OH)D_3$	401.50	383.25<br>365.30<br>159.00	-15<br>-15<br>-15	-9<br>-11<br>-31	-26<br>-25<br>-27
3-epi-$25(OH)D_3$	401.50	383.30<br>365.30<br>107.10	-15<br>-15<br>-12	-10<br>-11<br>-35	-27<br>-26<br>-17
$25(OH)D_2$	413.50	395.30<br>54.95<br>83.10	-10<br>-16<br>-12	-10<br>-52<br>-26	-18<br>-20<br>-30
3-epi-$25(OH)D_2$	413.50	395.30<br>54.95<br>83.10	-10<br>-10<br>-10	-10<br>-50<br>-25	-28<br>-21<br>-30
d_6-$25(OH)D_3$	407.30	389.10<br>371.20<br>158.90	-10<br>-10<br>-10	-9<br>-11<br>-25	-27<br>-18<br>-16

Table S2. Primers' sequences, annealing temperature, lengths of products obtained, and results of restriction in PCR reaction

Polymorphism	Starter sequences	Annealing temperature [°C]	PCR product length [bp]	Length of restriction fragments [bp]
CYP2R1 **(rs10741657)**	F: 5' GGGAAGAGCA ATGACATGGA 3'<br>R: 5' GCCCTGGAAG ACTCATTTTG 3'	51.8<br>51.8	287	GG: 151, 105 and 32<br>GA: 265, 151, 105 and 32<br>AA: 256, 32
CYP27A1 **(rs6709815)**	F: 5' GAATAGTACCCTCTGCATCCTCTGAGC 3'<br>R: 5' AGAACCCCAAACTTATCATGAGTTAGC 3'	61.3<br>56.7	712	GG: 475 and 237<br>GT: 712, 475 and 237<br>TT: 712
CYP27B1 **(rs10877012)**	F: 5' GCCTGTAGTG CCTTGAGAGG 3'<br>R: 5' CAGTGGGGAA TGAGGGAGTA 3'	55.9<br>53.8	187	GG: 138 and 49<br>GT: 187, 138 and 40<br>TT: 187

Figure S1. Chromatograms for a blank sample, LOQ, and volunteer's samples for the concentrations of (12 ng/mL, 2.9 ng/mL, 7 ng/mL, and 3.8 ng/mL for 25(OH)D_3, 3-epi-25(OH)D_3, 25(OH)D_2, and 3-epi-25(OH)D_2, respectively).

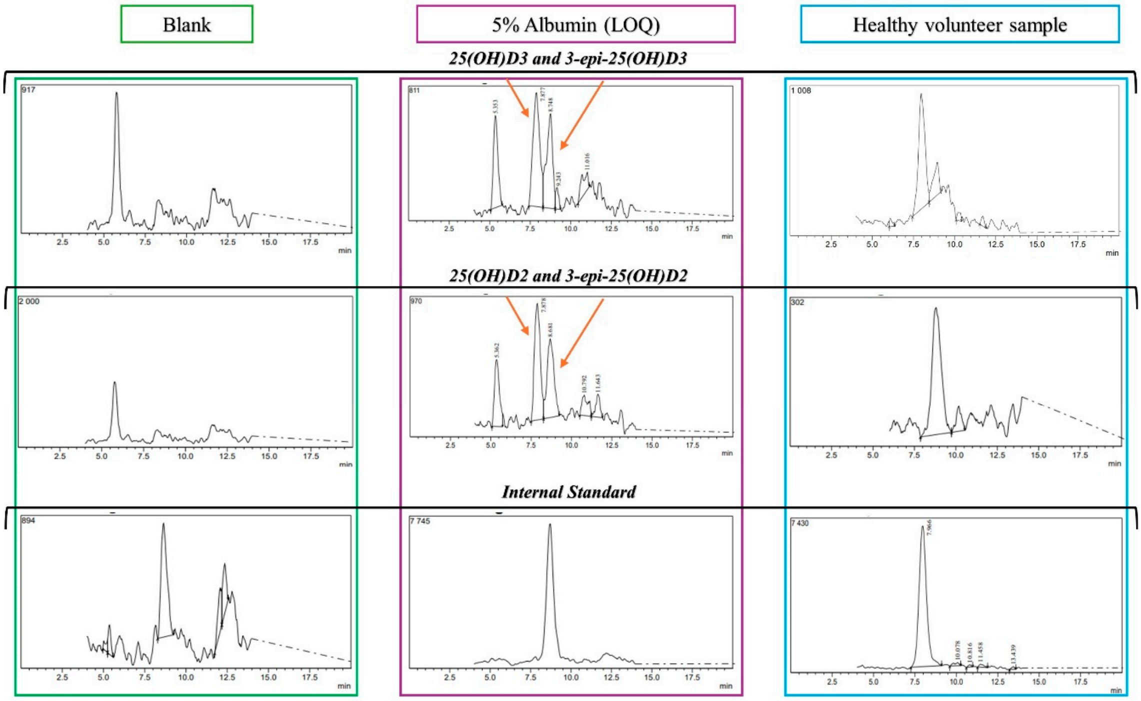

Supplement: Supplementary file 1 [file biomolecules-15-00699-s001.zip › biomolecules-3574957-supplementary.pdf]
